# Novel chimeric antigen receptors for the effective and safe treatment of NY‐BR‐1 positive breast cancer

**DOI:** 10.1002/ctm2.1776

**Published:** 2024-07-20

**Authors:** Dirk Jäger, Aileen Berger, Alexandra Tuch, Claudia Luckner‐Minden, Rosa Eurich, Mario Hlevnjak, Andreas Schneeweiss, Peter Lichter, Sebastian Aulmann, Claus‐Peter Heussel, Carlo Fremd, Richard Harbottle, Inka Zörnig, Patrick Schmidt

**Affiliations:** ^1^ Department of Medical Oncology, National Center for Tumor Diseases (NCT) University Hospital Heidelberg Heidelberg Germany; ^2^ Clinical Cooperation Unit “Applied Tumor Immunity” German Cancer Research Center (DKFZ) Heidelberg Germany; ^3^ Computational Oncology Group, Molecular Precision Oncology Program NCT and DKFZ Heidelberg Germany; ^4^ Division of Gynecological Oncology, NCT University Hospital Heidelberg Heidelberg Germany; ^5^ Division of Molecular Genetics DKFZ Heidelberg Germany; ^6^ NCT Heidelberg a partnership between DKFZ and Heidelberg University Medical Center Heidelberg Germany; ^7^ German Cancer Consortium (DKTK) Heidelberg Germany; ^8^ Institute for Pathology University Hospital Heidelberg Heidelberg Germany; ^9^ Department of Diagnostics and Interventional Radiology, Thoraxklinik Heidelberg University Hospital Heidelberg Heidelberg Germany; ^10^ DNA Vector Lab DKFZ Heidelberg Germany; ^11^ GMP and T Cell Therapy Group DKFZ Heidelberg Germany

Dear Editor,

Adoptive cellular immunotherapy is well‐established and has achieved very good outcomes in the treatment of leukemic diseases, however, the applicability of chimeric antigen receptor (CAR)‐Ts for the treatment of solid tumours remains very challenging mainly because of the lack of tumour‐restricted membranous antigens.^1^, ^2^ Here we report on the development of two novel breast cancer‐specific CARs that bind to NY‐BR‐1 (ANKRD30A) and show no cross‐reactivity with the brain tissue‐related isoform NY‐BR‐1.1.

First, we confirmed the frequency of NY‐BR‐1 expression and its localization, by performing in silico analyses across tumors on the latest The Cancer Genome Atlas (TCGA) Pan‐Cancer Atlas datasets.^3^ mRNA sequence data of ANKRD30A revealed the highest expression values in Breast Invasive Carcinoma and Prostate Adenocarcinoma (Figure 1A), although the latter contains approximately 10‐fold less data samples (833 vs. 89 log2 values > 2). For Breast Invasive Carcinoma, NY‐BR‐1 mRNA expression is not significantly correlated with tumour stage (Figure S1A), histomorphologic subtype (Figure S1B) or distant disease status (Figure S1C). However, the sample number for metastatic disease (M1) within the TCGA dataset has been low (*n* = 21). To overcome this limitation and to consider a clinically valuable trial population, we further analyzed NY‐BR‐1 expression in an institutional cohort of metastatic breast cancer (*n* = 180) accompanied by histopathological staining of another cohort (n = 139) (Figure S1D). Here, the highest median expression is prevalent in ER+ and HER2+ disease, well aligning with a slightly higher level of expression for lobular carcinoma in the publicly available dataset.^4^ To confirm the membrane‐bound expression of NY‐BR‐1,^5^ we have developed a binary FACS‐based scoring system using the commercial antibody clone2 on cell suspensions of breast cancer samples. Application of that scoring system identified 43 of 91 (47%) patients with surface expression of NY‐BR‐1, from which three samples showed a score of 2 (Figure 1C). In contrast, 71 patients (78%) stained positive for HER2 but without a correlation to NY‐BR‐1 status.

**FIGURE 1:**
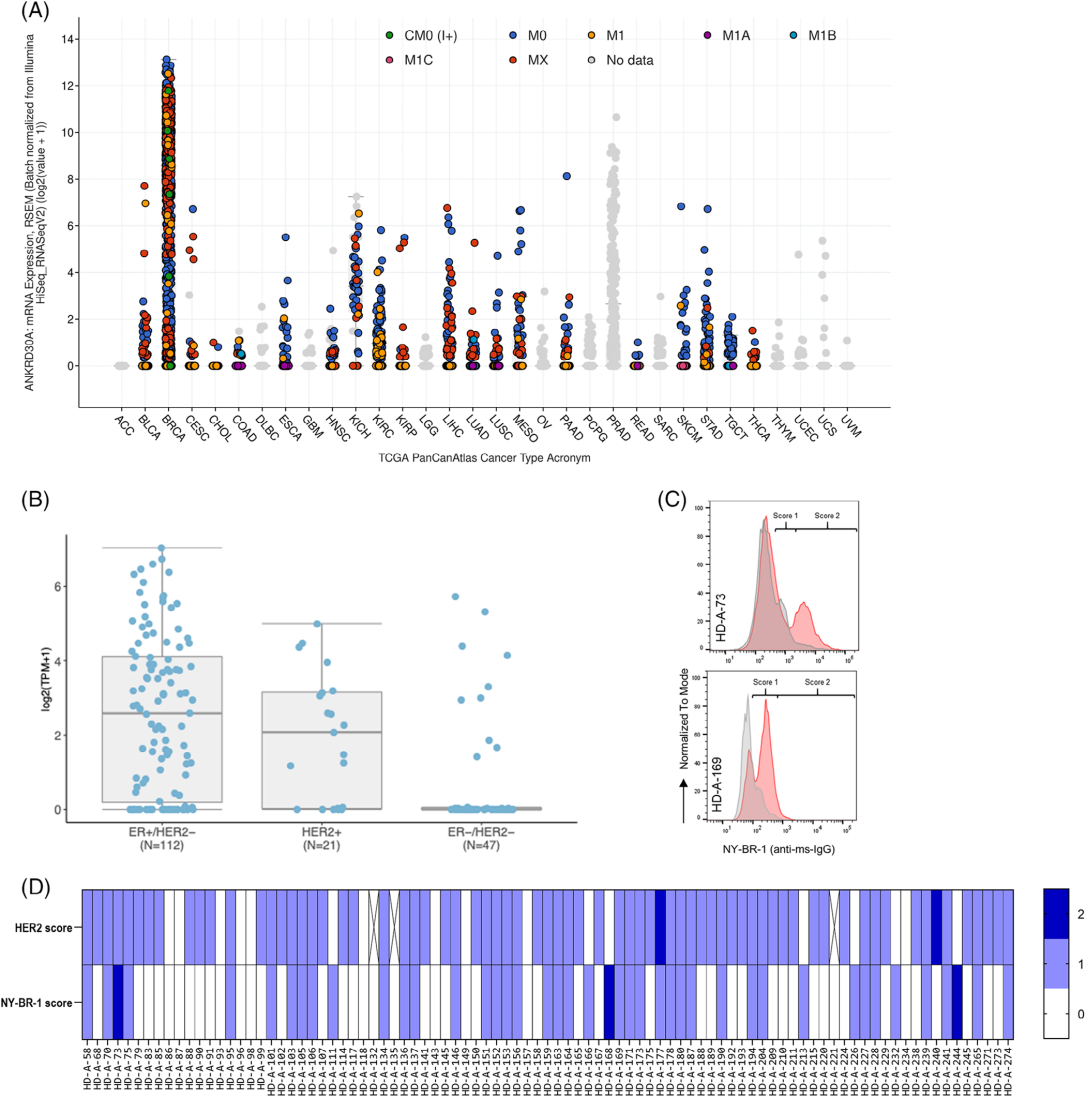
NY‐BR‐1 Expression analyses. (A) Normalized NY‐BR‐1 RNAseq data derived from The Cancer Genome Atlas (TCGA) datasets is shown, correlated with metastatic status (AJCC Metastasis Stage Code) and grouped by tumour entity (Study of Origin). Each dot represents a study sample, data was analyzed at cbioportal.org. (B) NY‐BR‐1 expression across intrinsic subtypes of an institutional, real‐world, metastatic breast cancer cohort (RNAseq, normalized by transcripts per kilobase million). (C) Representative FACS plot of two NY‐BR‐1 surface stainings using clone2 mAb on pleural effusion cells derived from breast cancer patients. Score1 indicates an MFI change to isotype control < 10‐fold, and Score2 indicates an MFI change to isotype control ≥10‐fold. (D) Combined scoring analysis of 91 primary breast cancer samples based on FACS stainings for HER2 and NY‐BR‐1 shows a positivity rate of 47.25% (43/91 samples) with respect to NY‐BR‐1 protein.

Next, we identified the binding epitope of clone2, a publicly available anti‐NY‐BR‐1 antibody, by the PEPperPRINT technology pipeline. By this method, we detected the peptide LKNEQTLRADQMF representing the specific epitope (Figure 2A). However, protein alignments and BLAST analyses through the whole exome of Homo sapiens revealed a highly similar peptide sequence with protein NY‐BR‐1.1 (ANKRD30B) that differs in only one amino acid from the discovered epitope. Additionally, we performed a bioinformatics analysis on public RNA scSeq datasets, which resulted in the detection of NY‐BR‐1.1 in neuronal cells of the brain as well as in spermatocytes (Figure 2B). In order to obtain more specific binders without cross‐reactivity we evaluated the binding epitopes of two novel antibodies named 10D11 and clone3 (a kind gift of the Ludwig Institute for Cancer Research, New York). For each of the antibodies, we detected a consensus epitope sequence, particularly RGQVRKLEKMTKR for 10D11 Ab and AEPPEKPSA for clone3 Ab (Figure 2C). We next evaluated whether both Abs and their respective scFv derivates bind to the membrane portion of cell line transfectants of NY‐BR‐1 and NY‐BR‐1.1 (Figure 2D). We found that both binders show a concentration‐dependent surface staining in serial dilution to NY‐BR‐1 while a complete absence of binding to NY‐BR‐1.1. To further analyze the cross‐reactivity of functional CAR‐T cells, we made use of full‐length protein‐coated plates, which were incubated with CAR‐T cells followed by ELISA‐based determination of interferon (IFN)gamma in the culture supernatant as a determinant of CAR‐T activation (Figure 2E). By that, we observed a significantly reduced activation of 10D11 and clone3 CAR‐T cells after encountering NY‐BR‐1.1 compared to NY‐BR‐1. Functionality experiments of lentivirally and pS/MARt DNA vector^6^ engrafted CAR‐T cells (Figure 2F) revealed an equal capacity for cytolysis (Figure 2G) and IFNgamma secretion (Figure 2H) among the three candidates.

**FIGURE 2:**
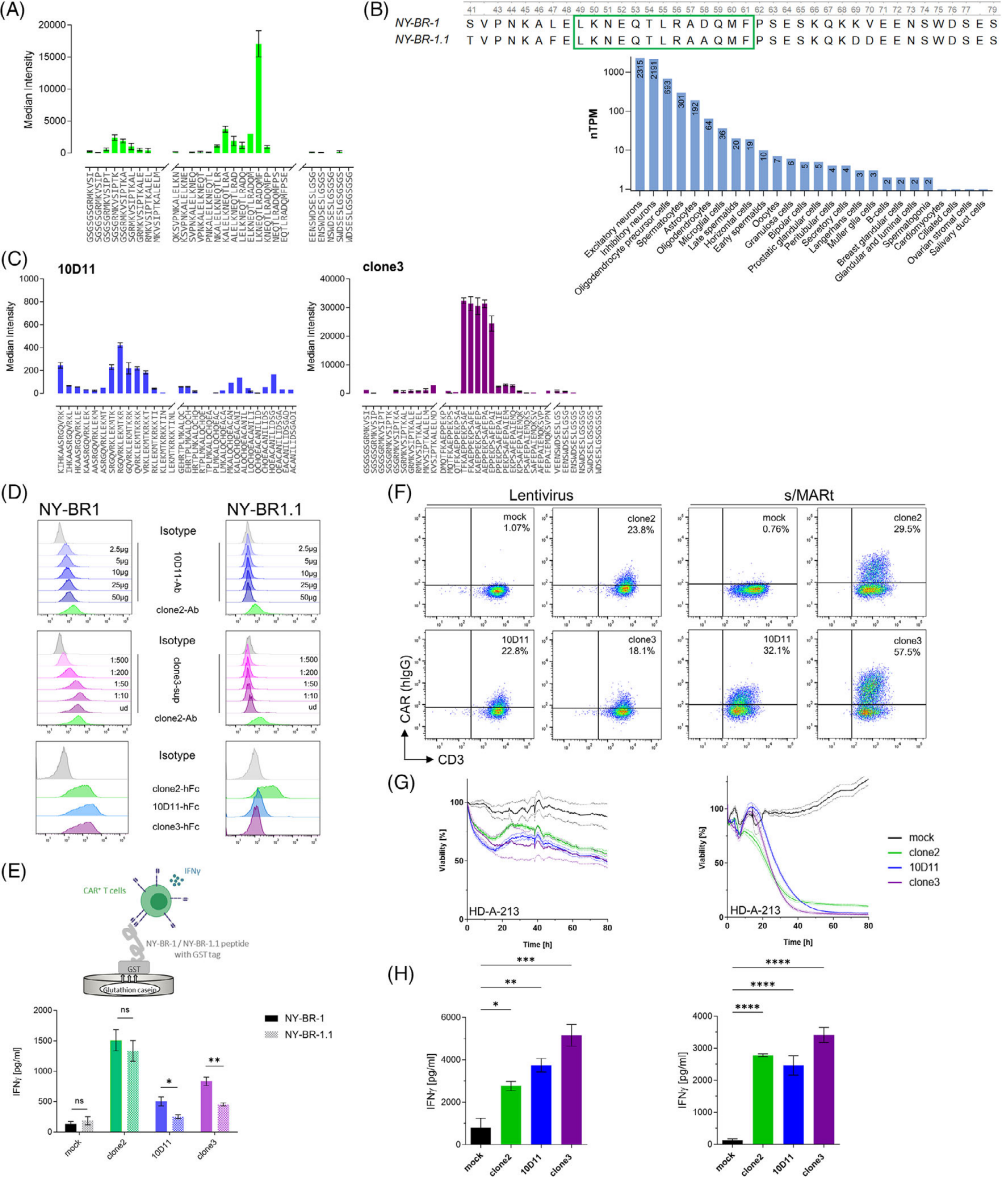
Cross‐reactivity investigations on new binders and in vitro functionality assays of anti‐NY‐BR‐1 chimeric antigen receptor (CAR)‐T cells. (A) PEPperMAP technology of overlapping peptide scanning exposes the clone2 epitope sequence. Shown here is the median fluorescence intensity of the secondary PE‐conjugated anti‐mouse Ab detecting clone2 mAb bound to a chip spotted with the indicated peptide sequences (left panel—each peptide was spotted on chip in triplicates). (B) Sequence alignment of the NY‐BR‐1 peptide used for clone2 mAb generation with NY‐BR‐1.1 full‐length protein reveals a highly similar sequence to the clone2 epitope in NY‐BR‐1.1. RNAseq data of NY‐BR‐1.1 derived from proteinatlas.org shows detectable NY‐BR‐1.1 signals in neuronal and sperm cells (lower panel). (C) Epitope discovery data of 10D11 and clone3 mAbs derived as in A is displayed. (D) FACS histogram plots of NY‐BR‐1 or −1.1 transfected BOSC23 cells incubated with serial dilutions of 10D11 and clone3 mAbs (purified mAb or hybridoma supernatant) followed by PE‐conjugated secondary anti‐mouse immunoglobulin G (IgG) Ab (upper two panels) or with fusion proteins in scFv‐Fc format (i.e. extracellular CAR domain) followed by PE‐conjugated secondary anti‐human IgG Ab. (E) Bar plot of interferon (IFN)γ secretion ELISA of supernatant derived from incubation of anti‐NY‐BR‐1 CAR‐T cells on protein‐coated plates (Mean ± SD n = 3; two‐tailed unpaired t‐test). (F) FACS plot images of CAR+ T cells 2 days after genetic engineering detected by the anti‐human IgG Ab. (G) Real‐time cytotoxicity assay of CAR‐T cells co‐cultivated with human pleural effusion cells from breast cancer patients. Measurement was done every 5 min with technical triplicates, dashed lines represent SD. (H) Bar plot of IFNγ ELISA derived from the supernatant of B at assay endpoint (Mean ± SD, one‐way analysis of variance [ANOVA] with multiple comparisons, Tukey corrected).

In order to analyse the in vivo functionality of the CAR constructs, we used BOSC23‐NY‐BR‐1 as a surrogate cell line for xenotransplantation assays. First, we treated tumour‐bearing mice with lentivirus CAR‐T cells and observed reduced tumour growth and prolonged survival (Figure 3A,B). This was accompanied by persistent CAR‐Ts and increased pro‐inflammatory cytokines in the blood (Figure S3). Unexpectedly, we were not able to replicate the result when treating mice with pS/MARt vector CAR T cells (Figure 3C,D and Figure S4) although a minor effect was observed. However, we found CAR‐Ts persistent in tumours and spleens as well as elevated inflammatory cytokines in blood post‐treatment (Figure 3E,F). This is opposed to lentiviral CAR‐T treatment experiments (Figure S3). Based on these results we speculated whether the local secretion of IL‐2 caused by endogenous CD28 signalling in combination with attraction of blocking macrophages in the tumor microenvironment might lead to a suppressive phenotype of pS/MARt CAR‐T cells.^7^, ^8^ Therefore, we introduced deleterious mutations to the FcR binding region of the IgG1 spacer domain and to the Lck binding region of the CD28 intracellular domain.^9^ Treatment of tumour‐bearing NSG mice with these mutated pS/MARt CAR‐Ts yielded a comparable outcome as seen with lentivirally transduced CAR‐Ts (Figure 3G,H and Figures S3 and S5).

**FIGURE 3:**
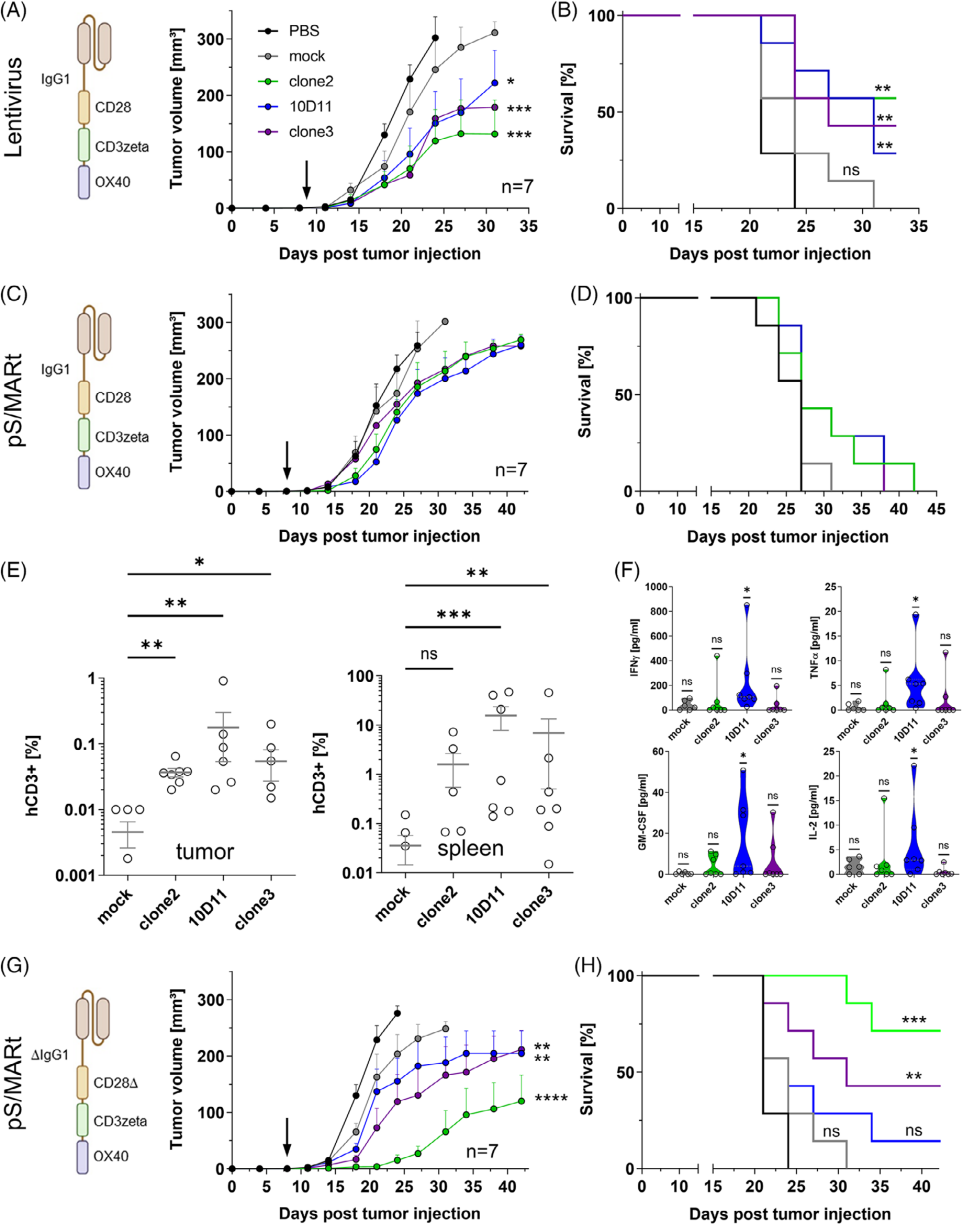
In vivo activity of NY‐BR‐1 directed chimeric antigen receptor (CAR)‐T cells in dependency of the mode of genetic engineering. (A) Tumour growth curves and (B) Kaplan‐Meier plot of mice bearing xenotransplanted tumour cells and treated with a single dose of lentivirally engineered CAR‐T cells. The arrow indicates the time of treatment, seven mice treated per group, and mean tumour growth plotted with SD (Ordinary two‐way analysis of variance [ANOVA] with Bonferroni corrected multiple comparisons). Survival proportions are plotted according to the number of subjects at risk (Log‐rank Mantel‐Cox test). (C) Mean tumour growth and (D) survival as in (A) for CAR‐T cells engineered with DNA vectors. (E) Scatter plots of FACS analyses from individual mice derived from (C). The amount of human CD3+ cells was determined in the suspension of tumour and splenic cells (*n* = 7; Kruskal‐Wallis test with uncorrected Dunn's test for multiple comparisons). (F) Violin plots of multiplexed cytokine measurement data of blood samples derived from (B) (*n* = 7; Wilcoxon signed‐rank test). (G) Mean tumour growth and (H) survival as in (A), for CAR‐T cells engineered with DNA vectors harbouring modified co‐stimulatory domains.

Finally, we analyzed our CAR‐T cells in a syngeneic NY‐BR1 transgenic mouse model (MMTV‐NY‐BR‐1^+/−^) for cross‐reactivity and persistence. We detected murine T cells in organs with high MMTV promotor activity, but not to a higher extent than in wild‐type animals (Figure 4B,C). However, a specific on‐target activation was observed in serum (Figure 4D) 24 h after injection but it did not cause pathological damage to the mice since there was no external sign of inflammation visible in either treatment group. We also utilized the model to test and compare our two novel binders within the previous effective OX40 format and the 4‐1BB format.^10^ Within the 4‐1BB setting, 10D11 and clone3 CAR‐Ts showed a higher rate of persistence in tg animals compared to wildtype as is the case with clone2 CAR‐Ts (Figure 4E), but cytokine secretion could not be detected (Figure 4F and Figure S6). This pinpoints to a higher activation threshold of 10D11 and clone3 which is favourable, as it needs target overexpression for effective attack.^11^ The OX40 format after all showed a mild splenic persistence of CAR‐Ts in tg and wt animals. Cytokine secretion of OX40‐based CARs also induced an off‐target in vivo effect in both mouse strains although not exceeding 4‐1BB CAR levels.

**FIGURE 4:**
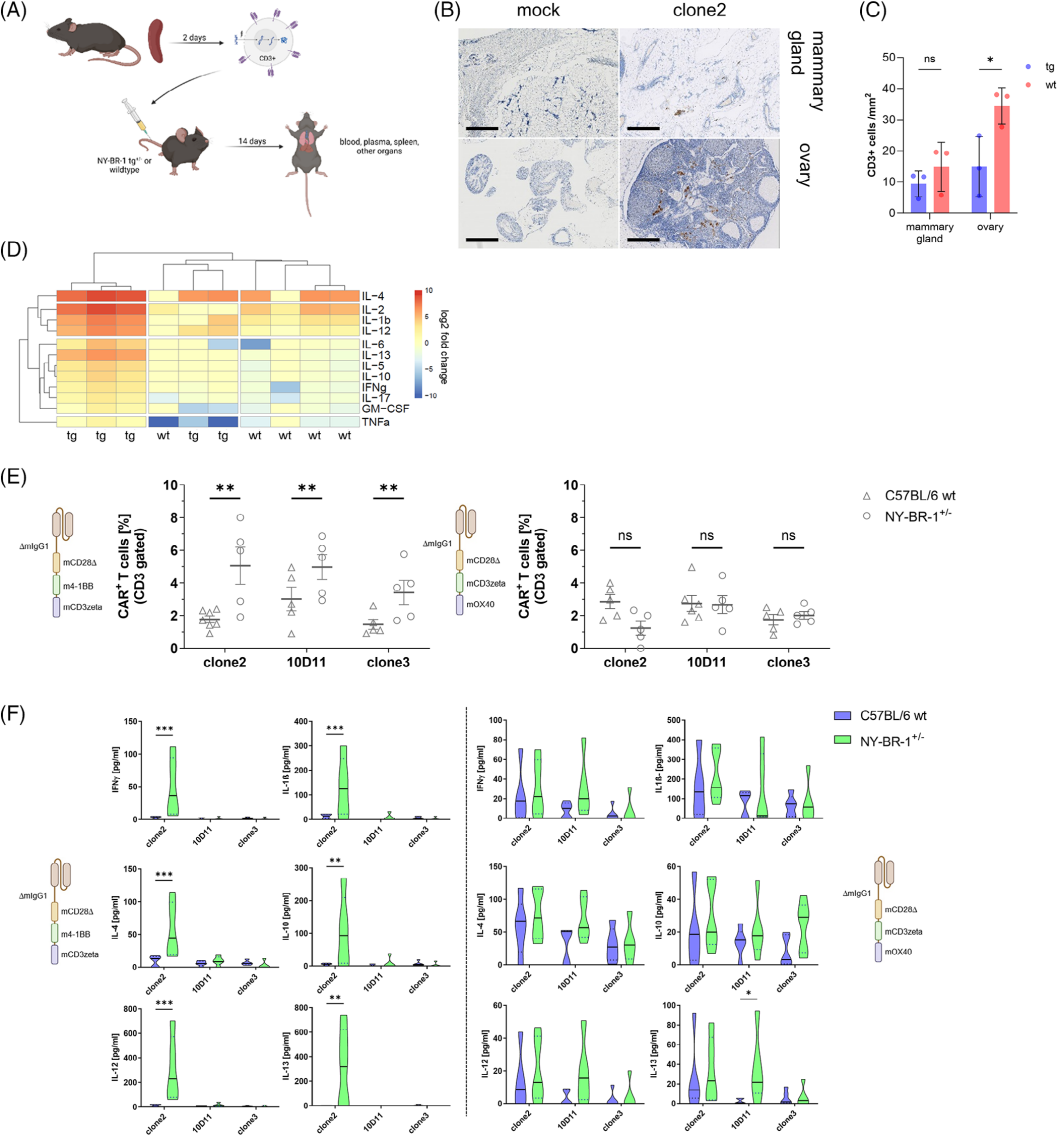
Safety investigations on anti‐NY‐BR1 chimeric antigen receptor (CAR)‐T cells. (A) Schematic overview of experimental design. (B) Representative IHC micrographs of CD3+ infiltration into affected organs at x40 magnification, the bar represents 500 μm. (C) Quantification of infiltrated T cells in CAR‐treated transgenic or wildtype mice determined by HALO software algorithm on tissue sections (*n* = 3 per tissue). (D) Heatmap of log2 fold change of multiplexed plasma cytokine measurements derived from transgenic and wildtype mice injected with clone2 CAR‐T cells, 24 h post‐injection. Non‐injected mice served as baseline control (*n* = 5, Euclidean distance measure with average linkage; Data analyzed using the ClustVis tool). (E) Scatter plots of FACS analyses from individual transgenic or wild‐type mice treated with syngeneic CAR‐T cells. The amount of CD3+/CAR+ cells was determined in suspension splenic cells (*n* = 5; Ordinary two‐way analysis of variance [ANOVA] with Bonferroni corrected multiple comparisons). (F) Violin plots of multiplexed cytokine measurement data of blood samples according to B (*n* = 5; Wilcoxon signed‐rank test).

Overall, we think that 10D11 and clone3‐based CAR‐T cells will be appropriate candidates for a Phase I clinical trial of anti‐NY‐BR‐1 cellular immunotherapy.

## AUTHOR CONTRIBUTIONS

Aileen Berger, Alexandra Tuch, Claudia Luckner‐Minden, Rosa Eurich, Sebastian Aulmann and Patrick Schmidt performed experiments. Dirk Jäger and Patrick Schmidt supervised the study. Dirk Jäger, Inka Zörnig, Carlo Fremd, Sebastian Aulmann, Mario Hlevnjak, Richard Harbottle analysed data. Dirk Jäger, Andreas Schneeweiss, Peter Lichter, Claus‐Peter Heussel and Richard Harbottle provided resources. Patrick Schmidt wrote the manuscript. Dirk Jäger, Inka Zörnig, Patrick Schmidt and Carlo Fremd revised the manuscript.

## CONFLICT OF INTEREST STATEMENT

Dirk Jäger, Inka Zörnig and Patrick Schmidt are owners and inventors on the patents WO2023/083982 and WO2023/083985 regarding the commercial use of CAR‐T cells targeting NY‐BR‐1.

## Supporting information

Supporting Information

Supporting Information

Supporting Information

Supporting Information

Supporting Information

Supporting Information
