# Supplementary figures and images for: Novel chimeric antigen receptors for the effective and safe treatment of NY‐BR‐1 positive breast cancer
Source: Clin Transl Med. 2024 Jul 20;14(7):e1776. doi: 10.1002/ctm2.1776 (PMC11260171; doi:10.1002/ctm2.1776)

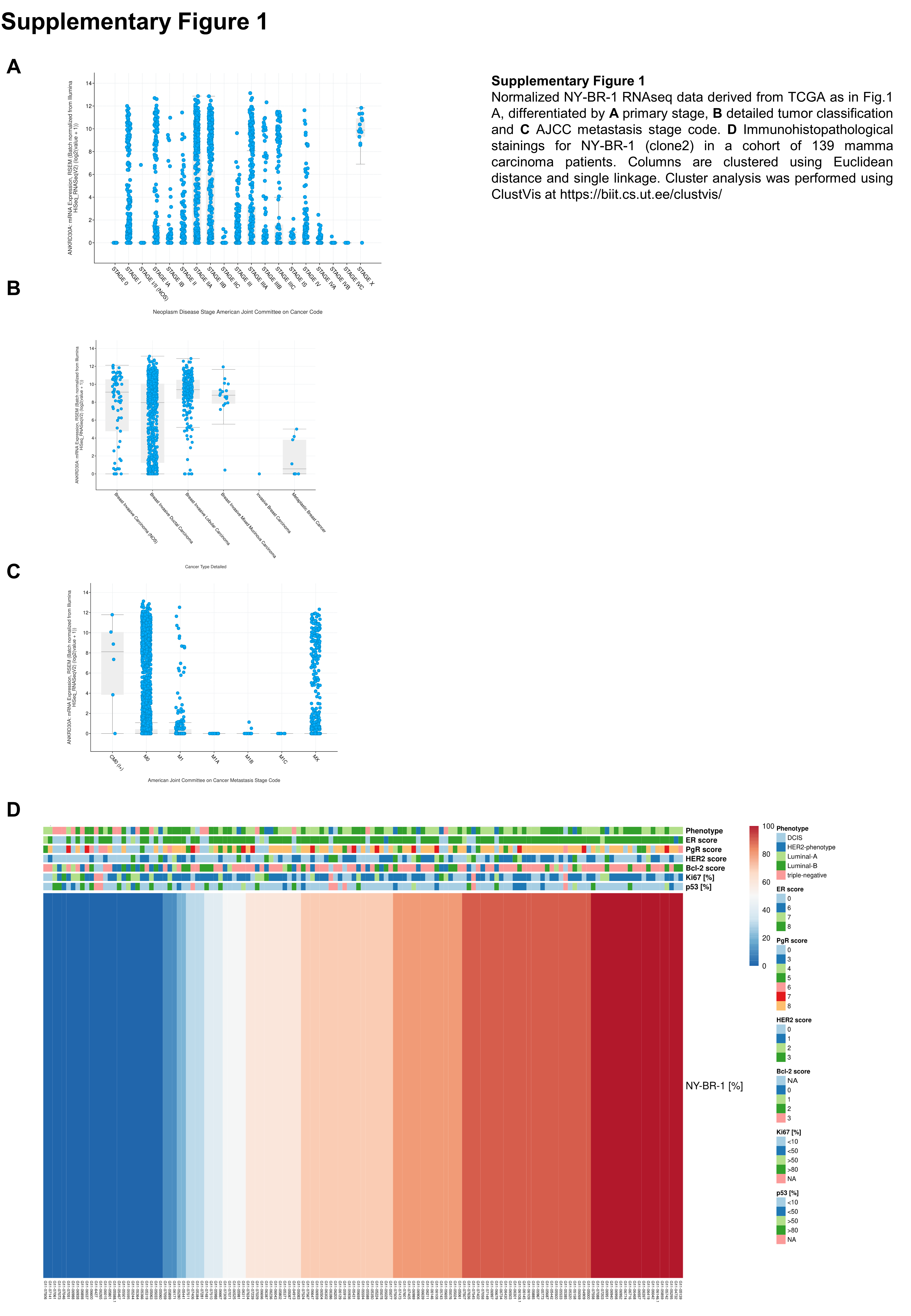

Supplement: Supplementary file 1 — Supporting Information [file CTM2-14-e1776-s006.tif]

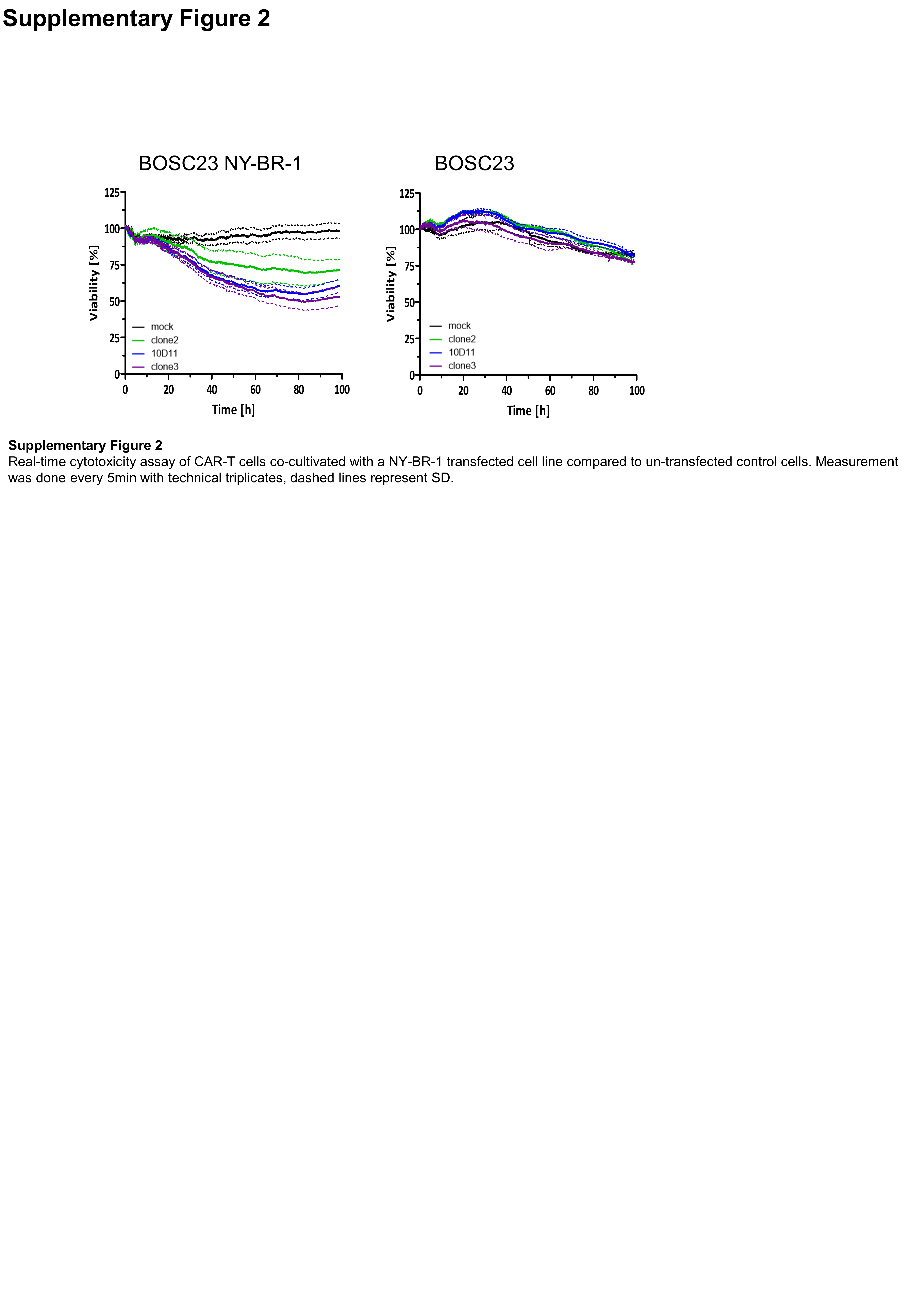

Supplement: Supplementary file 2 — Supporting Information [file CTM2-14-e1776-s002.tif]

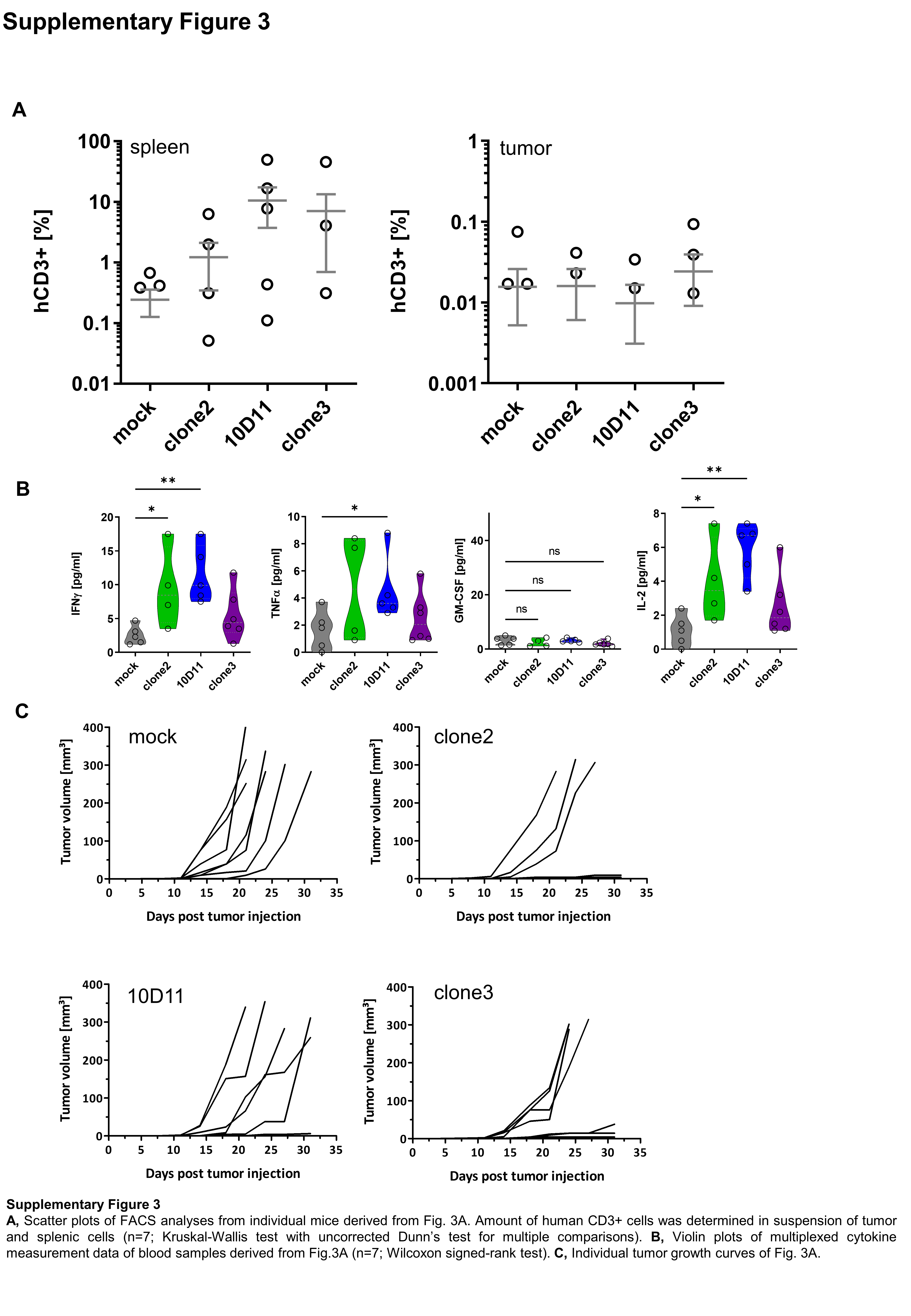

Supplement: Supplementary file 3 — Supporting Information [file CTM2-14-e1776-s001.tif]

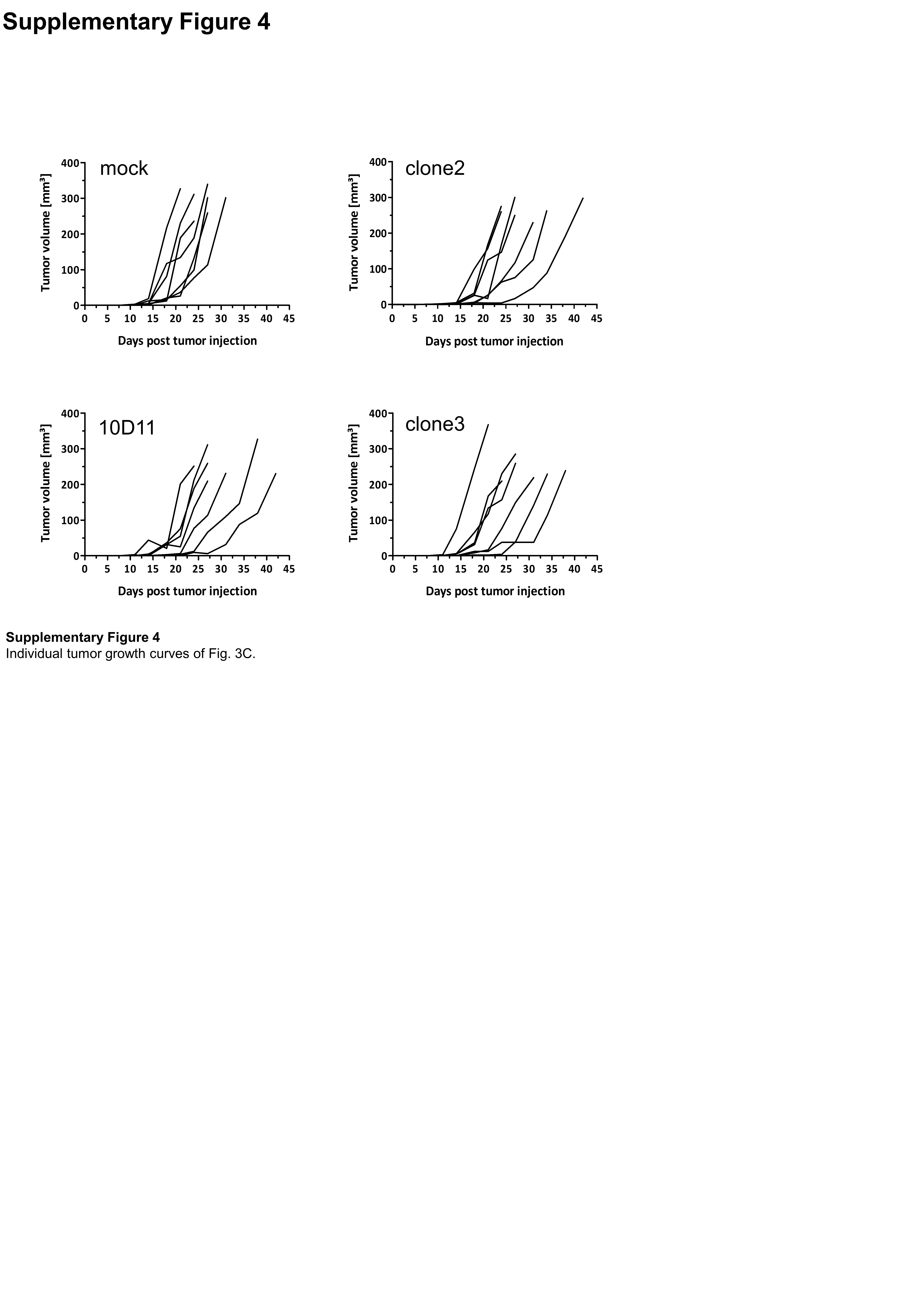

Supplement: Supplementary file 4 — Supporting Information [file CTM2-14-e1776-s003.tif]

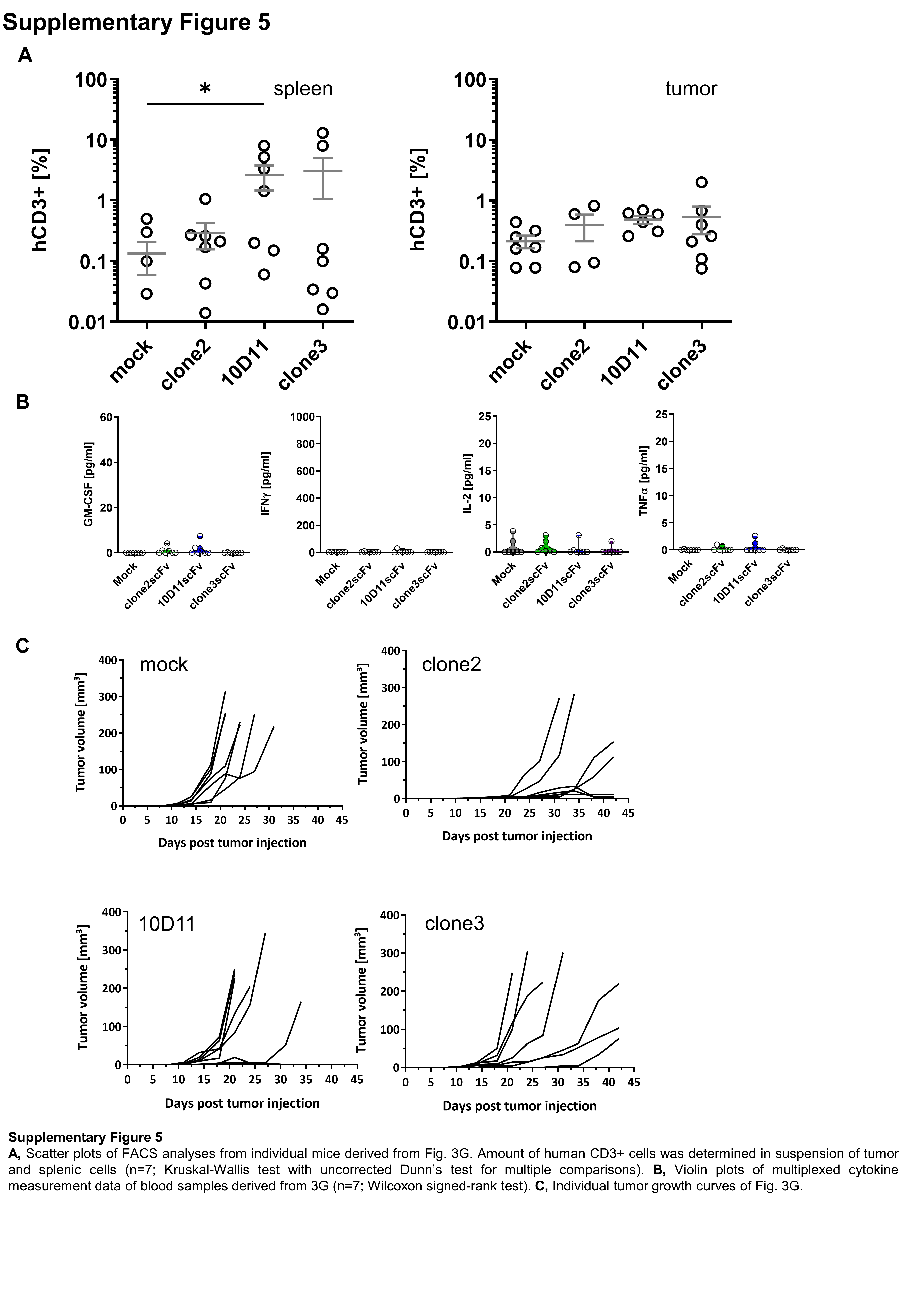

Supplement: Supplementary file 5 — Supporting Information [file CTM2-14-e1776-s004.tif]

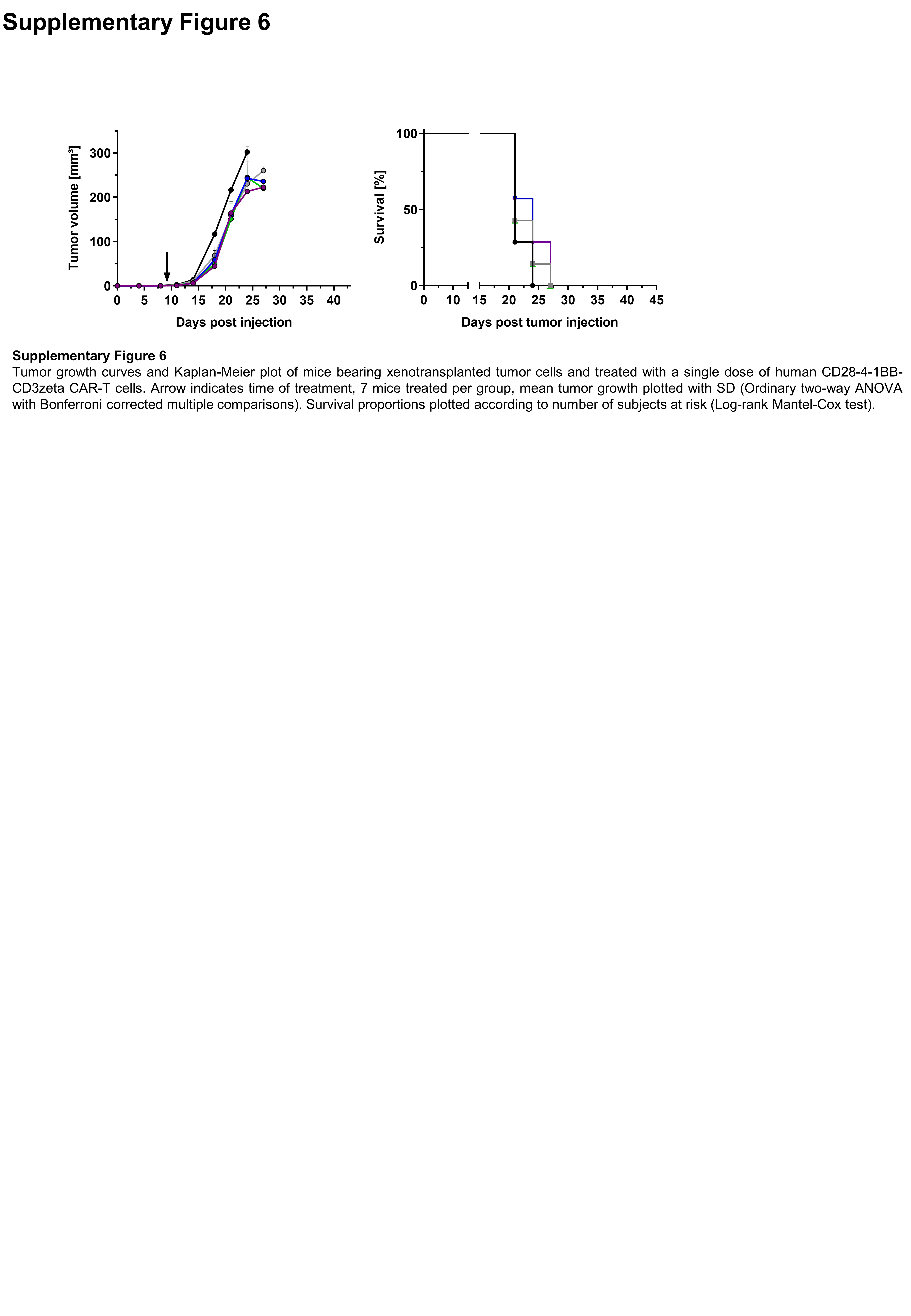

Supplement: Supplementary file 6 — Supporting Information [file CTM2-14-e1776-s005.tif]
